# Preoperative skeletal muscle index vs the controlling nutritional status score: Which is a better objective predictor of long‐term survival for gastric cancer patients after radical gastrectomy?

**DOI:** 10.1002/cam4.1548

**Published:** 2018-06-28

**Authors:** Zhi‐Fang Zheng, Jun Lu, Jian‐Wei Xie, Jia‐Bin Wang, Jian‐Xian Lin, Qi‐Yue Chen, Long‐Long Cao, Mi Lin, Ru‐Hong Tu, Chao‐Hui Zheng, Chang‐Ming Huang, Ping Li

**Affiliations:** ^1^ Department of Gastric Surgery Fujian Medical University Union Hospital Fuzhou China; ^2^ Department of General Surgery Fujian Medical University Union Hospital Fuzhou China; ^3^ Key Laboratory of Ministry of Education of Gastrointestinal Cancer Fujian Medical University Fuzhou China; ^4^ Fujian Key Laboratory of Tumor Microbiology Fujian Medical University Fuzhou China

**Keywords:** CONUT score, gastric cancer, long‐term survival, nutritional status, skeletal muscle index

## Abstract

Skeletal muscle index (SMI) and the controlling nutritional status (CONUT) score are useful for evaluating nutritional status, which is closely associated with cancer prognosis. This study compared the prognostic value of these indicators in patients with gastric cancer (GC) after radical gastrectomy (RG). We retrospectively enrolled 532 patients between 2010 and 2011. SMI was measured via CT images to determine low SMI. The CONUT score was calculated based on serum albumin, total lymphocyte count, and cholesterol. Patients were grouped according to SMI and the CONUT score based on previous research. Spearman's correlation coefficient, the Kaplan‐Meier method, and Cox regression were used. There was no significant correlation between SMI and the CONUT score. Five‐year overall survival (OS) and recurrence‐free survival (RFS) in patients with low SMI were significantly worse than those in patients with high SMI (*P* < .001). The normal nutrition group had better OS and RFS than did the light and moderate or severe malnutrition groups (*P* < .05), but the OS and RFS were not significantly different between the light and moderate or severe malnutrition groups (*P* = .726). Univariate analysis showed that SMI and the CONUT score were associated with OS and RFS, but only SMI remained prognostic in multivariate analysis. Preoperative SMI based on CT images is a more objective predictor than the CONUT score of long‐term survival in GC after RG, but this finding must be confirmed by prospective trials.

## 1 INTRODUCTION

Gastric cancer (GC) is the fourth most common malignancy and the second most common cause of cancer‐related deaths worldwide.^1^, ^2^ Therefore, accurate evaluation of prognosis in patients with GC may contribute to the development of individualized treatment programs and improve patient prognoses. Recently, nutritional status has been reported as a prognostic factor in patients with cancer.^3^, ^4^, ^5^, ^6^, ^7^, ^8^, ^9^

Sarcopenia, a syndrome characterized by progressive and generalized loss of skeletal muscle mass and strength, results in a decline in function, poor quality of life, and death.^10^, ^11^ Although weight can reflect nutritional status, sarcopenia (the loss of muscle mass) is a more accurate and quantitative indicator of frailty (nutritional status),^12^ and the effectiveness of sarcopenia (low skeletal mass index (SMI)) in predicting prognosis in GC has been widely documented.^5^, ^13^, ^14^, ^15^, ^16^ The CONUT score, derived from serum albumin (ALB), total lymphocyte count (TLC), and cholesterol measurements, is an effective tool for assessing the status of immune nutrition.^17^ The Controlling Nutritional Status (CONUT) score is a prognostic factor for various cancers, including GC.^8^, ^18^, ^19^, ^20^ Nevertheless, no studies have determined whether SMI or the CONUT score is a better predictor of long‐term prognosis in GC.

Therefore, the aim of this study was to compare the ability of preoperative SMI and the CONUT score to predict long‐term survival in GC after radical gastrectomy (RG).

## 2 MATERIALS AND METHODS

### 2.1 Materials

From a prospective database, 864 patients undergoing radical surgery for GC at Fujian Medical University Union Hospital (FMUUH) between 2010 and 2011 were identified. The exclusion criteria for this study were as follows: T4b patients (n = 31), intraoperative evidence of peritoneal tumor dissemination or distant metastasis (n = 9), patients with no available computed tomography (CT) images or with preoperative CT images older than 30 days (n = 235), incomplete clinical and pathologic data (n = 20), gastric stump carcinoma (n = 22), and preoperative neoadjuvant chemotherapy (n = 15). Ultimately, 532 patients were included in this study (Figure S1). Laboratory blood test data were collected within 1 week before surgery, including preoperative ALB and hemoglobin (HB) levels as well as lymphocyte counts and cholesterol concentrations. The type of surgical resection and the extent of lymph node dissection were selected according to the Japanese gastric cancer treatment guidelines,^21^ and the seventh corresponding edition of the American Joint Committee on Cancer (AJCC) Staging Manual was used to determine the disease stage.^22^ Patients with stage II‐III GC were advised to undergo adjuvant chemotherapy based on fluorine.^23^, ^24^ The study was approved by the FMUUH Institutional Review Board.

### 2.2 Measurement and grouping of SMI

Abdominal CT images were obtained from the computer center of the hospital, and skeletal muscle parameters were measured under the guidance of a professional radiologist. With Software Osirix version 3.3 (32‐bit; http://www.osirix-viewer.com),^25^ the third lumbar vertebra (L3) was set as a landmark, and two consecutive slices were selected to measure the cross‐sectional areas of the skeletal muscle. The mean value of two consecutive images was computed for each patient. The muscles in the L3 region include the rectus abdominis, psoas, quadratus lumborum, paraspinal, transverse abdominal, external oblique, internal oblique, and rectus abdominis muscles. Cross‐sectional skeletal muscle area was measured according to attenuation thresholds of −29 to +150 Hounsfield units (HU).^26^ Muscle areas were normalized for height (m^2^) to obtain the L3 SMI (cm^2^/m^2^).^27^ According to a previous study conducted by our center,^16^ 32.5 cm^2^/m^2^ for men and 28.6 cm^2^/m^2^ for women were defined as low SMI. Ultimately, 91 patients (17.1%) with low SMI and 441 patients with high SMI (82.9%) were enrolled in the study.

### 2.3 Definition and grouping of the CONUT score

The CONUT score was calculated based on serum ALB concentration, peripheral lymphocyte count, and peripheral cholesterol concentration (Table S1). Based on the total scores for the three parameters, nutritional status was categorized as normal nutrition, light malnutrition, moderate malnutrition, or severe malnutrition.^17^ Because we identified only four patients with severe malnutrition in our study, we integrated moderate and severe malnutrition into a single CONUT group for all subsequent analyses.^18^ Ultimately, 291 patients (54.7%) were included in the normal nutrition group, 183 patients in the light malnutrition group (34.4%), and 58 patients in the moderate or severe malnutrition group (10.9%).

### 2.4 Follow‐up

All the patients were followed up by telephone interview, outpatient visits, and letters. All surviving patients were followed up for more than 5 years. All patients were monitored postoperatively by physical examination and laboratory tests, including tests for tumor markers (such as carcinoembryonic antigen (CEA) and carbohydrate antigenic determinant (CA) 19‐9), every 3 months for the first 2 years, every 6 months for the next 3 years, and annually thereafter. In addition, examinations, including chest radiography, abdominopelvic CT, and endoscopy, were performed at least once per year. If necessary, further evaluations, such as positron emission tomography or magnetic resonance imaging, were initiated to better identify recurrence.

### 2.5 Statistical analysis

Statistical analyses were performed with SPSS software, version 18.0 (SPSS Inc., Chicago, IL, USA), and R software, version 3.1.2 (R Foundation for Statistical Computing, Vienna, Austria). The significance tests used were Student's *t* test for continuous variables and the chi‐square test or Fisher's exact test for categorical variables. The relationships among studied parameters were examined using Spearman's correlation coefficient. A correlation was considered weak with coefficient values <0.5 and strong with values >0.8. The Kaplan‐Meier method was used to analyze overall survival (OS) and recurrence‐free survival (RFS), and the differences were assessed with log‐rank tests. A Cox proportional‐hazard model was used to identify variables with significant independent relationships with OS and RFS. Two‐tailed *P* values <.05 were considered statistically significant.

## 3 RESULTS

### 3.1 Clinicopathological data

The clinical and pathological data of the patients are shown in Table 1. Age, female sex, tumor size, tumor‐node‐metastasis (TNM) stage, comorbidities, and American Society of Anesthesiologists (ASA) status were all significantly higher in patients with low SMI than in those with high SMI. BMI, HB, ALB, and lymphocyte count were significantly lower in patients with low SMI than in those with high SMI. Conversely, SMI was not affected by tumor site, histological type, cholesterol concentration, operation method, type of resection, type of reconstruction, surgical duration, intraoperative blood loss, neurovascular invasion, or adjuvant chemotherapy. There were significant differences in age, BMI, tumor size, TNM, HB, ALB, lymphocyte count, cholesterol concentration, ASA, type of resection, and intraoperative blood loss in patients with different nutritional statuses. However, there were no significant differences in tumor site, histological type, comorbidities, operation method, type of reconstruction, neurovascular invasion, or adjuvant chemotherapy.

**Table 1:** Clinicopathological characteristics of 532 patients with gastric cancer undergoing radical gastrectomy

Variable	All	SMI			COUNT score			
		Low (n = 91)	High (n = 441)	*P* value	Normal (n = 291)	Light (n = 183)	Moderate or severe (n = 58)	*P* value
Age (y)	61.1 (11.5)	68.4 (11.4)	59.6 (10.9)	<.001	58.8 (11.1)	62.5 (11.4)	67.7 (10.3)	<.001
Gender								
Female	129 (24.2)	41 (45.1)	88 (20.0)	<.001	72 (24.7)	36 (19.7)	21 (36.2)	.036
Male	403 (75.8)	50 (54.9)	353 (80.0)		219 (75.3)	147 (80.3)	37 (63.8)	
BMI (kg/m^2^)	21.9 (3.5)	20 (2.7)	22.3 (3.5)	<.001	22.3 (3.5)	21.3 (2.9)	21.7 (4.6)	.012
Tumor site								
Upper	159 (29.9)	27 (29.7)	132 (29.9)	.96	79 (27.1)	65 (35.5)	15 (25.9)	.119
Not upper	373 (70.1)	64 (70.3)	309 (70.1)		212 (72.9)	118 (64.5)	43 (74.1)	
Tumor size (cm)	4.5 (2.5)	3.2 (2.0)	4.8 (2.5)	<.001	4.8 (2.5)	4.1 (2.3)	4.4 (2.7)	.006
TNM stage								
I	165 (31)	16 (17.6)	149 (33.8)	.001	111 (38.1)	45 (24.6)	9 (15.5)	.001
II	123 (23.1)	17 (18.7)	106 (24.0)		63 (26.6)	46 (25.1)	14 (24.1)	
III	244 (45.9)	58 (63.7)	186 (42.2)		117 (40.2)	92 (50.3)	35 (60.3)	
Histological type								
Differentiated	156 (29.3)	21 (23.1)	135 (30.6)	.151	88 (30.2)	50 (27.3)	18 (31)	.758
Undifferentiated	376 (70.7)	70 (76.9)	306 (69.4)		203 (69.8)	133 (72.7)	40 (69)	
Comorbidities								
No	390 (73.3)	55 (60.4)	335 (76)	.002	217 (74.6)	133 (72.7)	40 (69)	.659
Yes	142 (26.7)	36 (39.6)	106 (24.0)		74 (25.4)	50 (27.3)	18 (31)	
HB (g/dL)	12.5 (2.1)	11.4 (2.6)	12.8 (2.5)	<.001	13.5 (2.1)	12.0 (2.3)	9.2 (2.5)	<.001
ALB (g/dL)	3.8 (0.5)	3.7 (06)	3.9 (0.5)	.004	4.1 (0.3)	3.7 (0.4)	2.9 (0.5)	<.001
Lymphocyte (mm^3^)	1770 (620)	1530 (680)	1820 (600)	<.001	2020 (530)	1540 (610)	1280 (510)	<.001
Cholesterol (mg/dL)	189.5 (85.8)	182.7 (53.4)	190.9 (91.7)	.405	208.1 (37.6)	178.0 (130.8)	131.9 (34.9)	<.001
ASA								
I	202 (38)	19 (20.9)	183 (41.5)	.001	130 (44.7)	66 (36.1)	6 (10.3)	<.001
II	312 (59.2)	69 (75.8)	246 (55.8)		154 (52.9)	111 (60.7)	50 (86.2)	
III	15 (2.8)	3 (3.3)	12 (2.7)		7 (2.4)	6 (3.3)	2 (3.4)	
Operation method								
Open	76 (14.3)	15 (16.5)	61 (13.8)	.51	36 (12.4)	30 (16.4)	10 (17.2)	.327
Laparoscopic	456 (85.7)	76 (83.5)	380 (86.2)		255 (87.6)	153 (83.6)	48 (82.9)	
Type of resection								
Subtotal gastrectomy	297 (55.8)	49 (53.8)	248 (56.2)	.676	176 (60.5)	88 (48.1)	33 (56.9)	.03
Total gastrectomy	235 (44.2)	42 (46.2)	193 (43.8)		115 (39.5)	95 (51.9)	25 (43.1)	
Type of reconstruction								
Billroth I	250 (47)	41 (45.1)	209 (47.6)	.776	150 (51.5)	73 (39.9)	27 (46.6)	.22
Billroth II	34 (6.4)	8 (8.8)	26 (5.9)		16 (5.5)	14 (7.7)	4 (6.9)	
Roux‐en‐Y	235 (44.2)	40 (44)	195 (44.2)		116 (39.9)	93 (50.8)	26 (44.8)	
Other	13 (2.4)	2 (2.1)	11 (2.5)		9 (3.1)	3 (1.6)	1 (1.7)	
Surgical durations	189.5 (85.8)	1856.0 (67.2)	183.4 (58.1)	.706	177.5 (56.9)	191.9 (64.8)	190.0 (53.8)	.027
Intraoperative blood loss	96.2 (192.3)	79.1 (66.5)	99.7 (208.9)	.353	81.6 (110.1)	98.6 (132.3)	161.6 (470.5)	.015
Neurovascular invasion								
No	400 (75.2)	64 (70.3)	336 (76.2)	.239	229 (78.7)	129 (70.5)	42 (72.4)	.115
Yes	132 (24.8)	27 (29.7)	105 (23.8)		62 (21.3)	54 (29.5)	16 (27.6)	
Adjuvant chemotherapy								
No	280 (52.6)	46 (50.5)	234 (53.1)	.662	157 (54.0)	92 (50.3)	31 (53.4)	.731
Yes	252 (47.4)	45 (49.5)	207 (46.9)		134 (46.0)	91 (49.7)	27 (46.4)	

ALB, albumin; ASA, American Society of Anesthesiologists; BMI, body mass index; COUNT, controlling nutritional status; HB, hemoglobin; SMI, skeletal muscle index.

### 3.2 Correlation analysis

Spearman's correlation analysis showed weak correlations of SMI with ALB, lymphocyte count, and cholesterol (all *r*_s _< 0.5) (Table 2).

**Table 2:** Correlation between measurements of preoperative SMI and CONUT scores in patients with gastric cancer

Correlation coefficient (Spearman's p)	SMI
ALB	0.136
Lymphocyte	0.272
Cholesterol	0.033

ALB, albumin; COUNT, controlling nutritional status; SMI, skeletal muscle index.

### 3.3 SMI, CONUT score and survival

**Figure 1:**
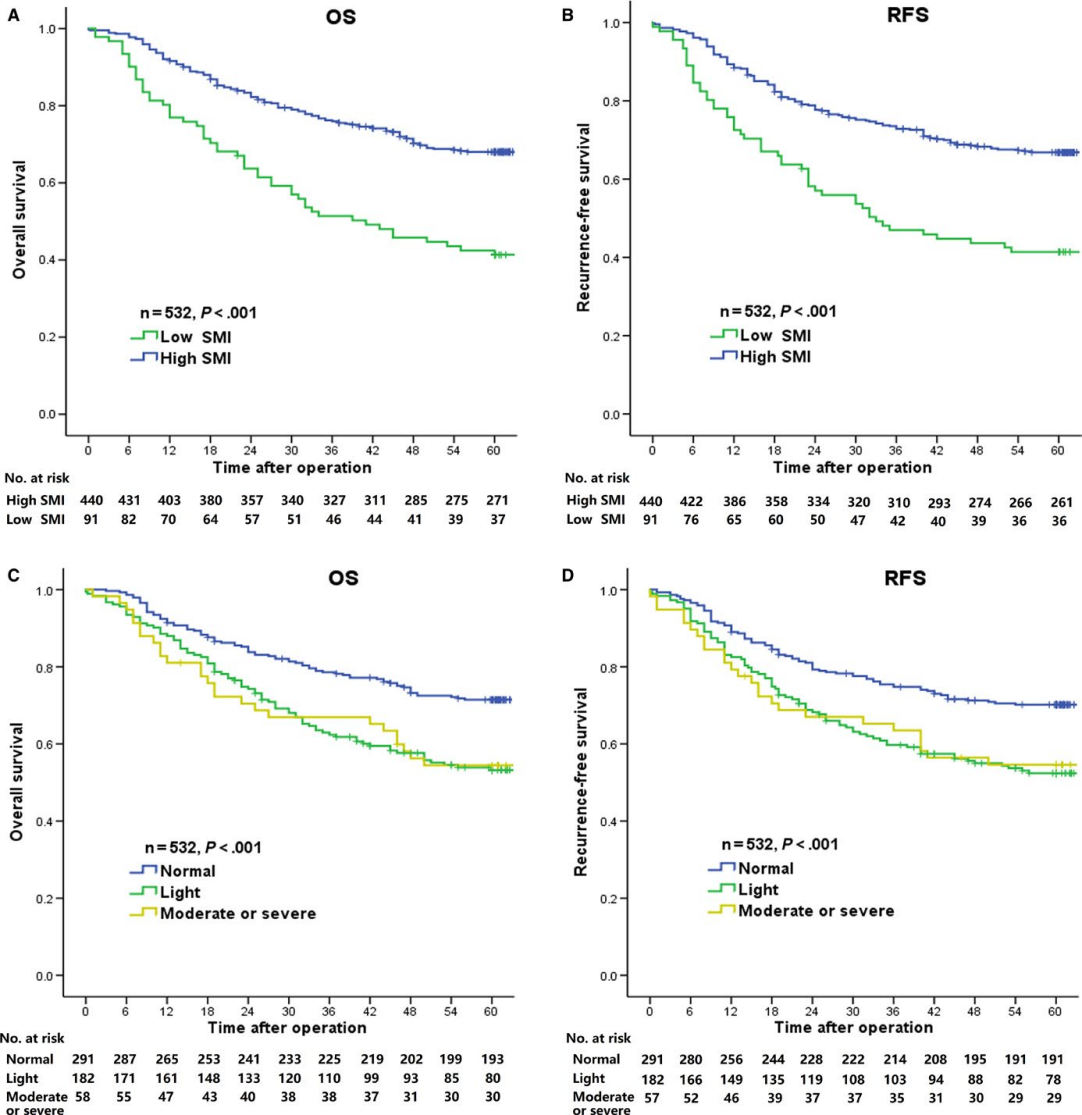
Kaplan‐Meier survival curves for overall survival (OS) according to SMI (A) and the CONUT score (C); Kaplan‐Meier survival curves for recurrence‐free survival (RFS) according to SMI (B) and the CONUT score (D)

The median duration of follow‐up was 60 months (range 2‐76 months). The 5‐year OS and RFS after surgery in patients with low SMI were significantly worse than those in patients with high SMI (41.30% vs 68%, *P* < .001; 42.60%, vs 66.2%, *P* < .001). Patients with normal nutrition had better 5‐year OS and RFS than did those with light malnutrition (71.40% vs 53.20%, *P* < .001; 70% vs 51.60%, *P* < .001), and these metrics were also better than those in patients with moderate or severe malnutrition (71.40% vs 54.50%, *P* = .006; 70% vs 55.20%; *P* = .017). However, there were no significant differences in 5‐year OS and RFS between patients with light malnutrition and those with moderate or severe malnutrition (*P* = .726) (Figure 1).

In univariate analysis, SMI, the CONUT score, age, tumor site, TNM, HB, ASA, type of resection, type of reconstruction, surgical duration, neurovascular invasion, and adjuvant chemotherapy were associated with 5‐year OS. Regarding 5‐year RFS, univariate analysis showed that SMI, the CONUT score, age, tumor site, TNM, histological type, type of resection, type of reconstruction, surgical duration, neurovascular invasion, and adjuvant chemotherapy were significantly associated (Table 3). In multivariate analysis, only TNM and SMI were independent prognostic factors for 5‐year OS and RFS (Table 3).

**Table 3:** Uni‐ and multivariate analyses of factors associated with 5‐year overall survival (OS) and recurrence‐free survival (RFS) rates in patients with gastric cancer

Variable	Univariate analysis 5‐year OS		Multivariate analysis 5‐year OS		Univariate analysis 5‐year RFS		Multivariate analysis 5‐year RFS	
	HR (95% CI)	*P*	HR (95% CI)	*P*	HR (95% CI)	*P*	HR (95% CI)	*P*
SMI								
High	Reference	<.001	Reference	.002	Reference	<.001	Reference	.012
Low	2.337 (1.702‐2.309)		1.704 (1.209‐2.403)		2.125 (1.545‐2.925)		1.553 (1.101‐2.189)	
COUNT Score								
Normal	Reference	<.001	Reference	.173	Reference	<.001	Reference	.137
Light	1.856 (1.367‐2.519)		1.360 (0.984‐1.879)		1.837 (1.362‐2.478)		1.376 (1.005‐1.884)	
Moderate or severe	1.839 (1.183‐2.859)		1.266 (0.753‐2.126)		1.703 (1.091‐2.659)		1.154 (0.726‐1.836)	
Age (y)								
<65	Reference	.002	Reference	.766	Reference	.006	Reference	.473
≥65	1.563 (1.176‐2.078)		1.054 (0.733‐1.517)		1.486 (1.123‐1.968)		1.124 (0.817‐1.547)	
Gender								
Female	Reference	.131			Reference	.051		
Male	0.783 (0.571‐1.075)				0.736 (0.541‐1.002)			
BMI (kg/m^2^)								
<25	Reference	.086			Reference	.156		
≥25	0.654 (0.402‐1.063)				0,716 (0.451‐1.136)			
Tumor site								
Upper	Reference	.008	Reference	.303	Reference	0.005	Reference	.103
Not upper	0.673 (0.501‐0.903)		0.824 (0.569‐1.192)		0.657 (0.491‐0.878)		0.736 (0.509‐1.064)	
Tumor size (cm)								
<5.0	Reference	.076			Reference	0.100		
≥5.0	0.768 (0.573‐1.028)				0.786 (0.591‐1.047)			
TNM stage								
I	Reference	<.001	Reference	<.001	Reference	<.001	Reference	<.001
II	2.125 (1.165‐3.876)		1.931 (1.042‐3.576)		2.260 (1.267‐4.030)		2.029 (1.120‐3.678)	
III	8.331 (5.009‐13.612)		7.520 (4.457‐12.690)		8.178 (5.065‐13.202)		7.337 (4.365‐12.334)	
Histological type								
Differentiate	Reference	.063			Reference	.026	Reference	.784
Undifferentiated	1.362 (0.984‐1.884)				1.447 (1.045‐2.004)		0.953 (0.677‐1.342)	
Comorbidities								
No	Reference	.352			Reference	.555		
Yes	1.160 (0.849‐1.583)				1.098 (0.806‐1.496)			
HB (g/L)								
>90	Reference	.036	Reference	.702	Reference	.056		
≤90	1.535 (1.028‐2.292)		0.911 (0.564‐1.471)		1.477 (0.990‐2.204)			
ASA								
I	Reference	.044	Reference	.435	Reference	.102		
II	1.483 (1.089‐2.021)		1.277 (0.880‐1.854)		1.377 (1.018‐1.863)			
III	1.395 (0.602‐3.231)		1.189 (0.480‐2.945)		1.501 (0.688‐3.278)			
Operation method								
Open	Reference	.173			Reference	.218		
Laparoscopic	0.768 (0.525‐1.123)				0.788 (0.540‐1.151)			
Type of resection								
Subtotal gastrectomy	Reference	<.001	Reference	.802	Reference	<.001	Reference	.944
Total gastrectomy	1.944 (1.460‐2.591)		1.089 (0.559‐2.123)		1.827 (1.379‐2.421)		1.023 (0.538‐1.945)	
Type of reconstruction								
Billroth I	Reference	<.001	Reference	.191	Reference	<.001	Reference	.225
Billroth II	2.582 (1.510‐4.414)		1.843 (1.044‐3.254)		2.562 (1.522‐4.313)		1.745 (1.008‐3.019)	
Roux‐en‐Y	2.124 (1.555‐2.901)		1.158 (0.568‐2.362)		2.042 (1.505‐2.770)		1.077 (0.544‐2.132)	
Other	1.199 (0.436‐3.294)		0.926 (0.314‐2.735)		1.122 (0.409‐3.077)		0.892 (0.307‐2.590)	
Surgical durations (min)								
<180	Reference	.007	Reference	.237	Reference	.005	Reference	.189
≥180	1.502 (1.118‐2.019)		1.204 (0.885‐1.637)		1.519 (1.135‐2.032)		1.224 (0.905‐1.656)	
Intraoperative blood loss (mL)								
<50	Reference	0.380			Reference	.342		
≥50	1.402 (0.659‐2.983)				1.442 (0.678‐3.066)			
Neurovascular invasion								
No	Reference	.008	Reference	.442	Reference	.007	Reference	.406
Yes	1.523 (1.119‐2.074)		1.133 (0.824‐1.557)		1.516 (1.118‐2.057)		1.141 (0.836‐1.558)	
Adjuvant chemotherapy								
No	Reference	.036	Reference	.062	Reference	.005	Reference	.281
Yes	1.357 (1.021‐1.084)		0.747 (0.552‐1.012)		1.492 (1126‐1.976)		0.849 (0.631‐1.143)	

ASA, American Society of Anesthesiologists; BMI, body mass index; CI, confidence interval; COUNT, controlling nutritional status; HB, hemoglobin; HR, hazard ratio; OS, overall survival; RFS, recurrence‐free survival; SMI, skeletal muscle index.

## 4 DISCUSSION

The determinants of cancer progression and prognosis are multifaceted, and increasing attention has been paid to the relationship between cancer and malnutrition.^3^, ^28^ Over the past few decades, malnutrition has been associated with a poor response to treatment, decreased quality of life, a higher risk of chemotherapy side effects, and worse prognosis.^4^, ^5^, ^29^, ^30^

CT imaging to assess body composition has been widely used in the field of cancer treatment and research due to its universality, high accuracy, and low incremental costs. Sarcopenia (low SMI), a multifactorial clinical condition, is closely associated with nutritional deficiencies.^5^, ^31^ After analyzing the survival data of 937 GC patients with TNM stage II or III who underwent RG, Zhuang et al^13^ concluded that sarcopenia (low SMI) was an independent risk factor for OS and RFS. Kensuke's studies suggested that sarcopenia (low SMI) was associated with a negative prognosis in esophagogastric junction cancer or upper GC.^14^ In addition, previous studies in our center have demonstrated that combining sarcopenia (low SMI) with the cT and cN system could accurately predict long‐term survival after RG for GC.^16^

The CONUT score has been established as a useful tool to evaluate nutritional status,^17^ and it is closely related to the prognosis of various cancers.^18^, ^19^, ^20^ The CONUT score not only reflects the nutritional status of patients with GC but also predicts long‐term OS after surgery for GC.^8^ Takagi et al^19^ suggested that the CONUT score was a reliable predictor of long‐term prognosis after hepatectomy for hepatocellular carcinoma. In addition, the predictive ability of the CONUT score is better than that of classic indicators, such as the neutrophil to lymphocyte ratio (NLR), prognostic nutritional index (PNI), and modified Glasgow Prognostic Score (mGPS).^8^, ^19^ However, whether the predictive power of the CONUT score is superior to that of sarcopenia has not been previously reported.

In this study, the CONUT score and SMI were prognostic factors for OS and RFS after RG according to univariate analysis, but in multivariate analysis, only SMI remained an independent prognostic factor for OS and RFS. Although Kuroda et al^8^ found that the CONUT score was an independent risk factor for long‐term survival after surgery for GC and was superior to NLR and mGPS, it was not included among the factors for SMI. The present study included both the CONUT score and SMI and revealed that the prognostic ability of SMI was better than that of the CONUT score. The possible reasons for this finding are as follows. The CONUT score is calculated based on plasma ALB concentration, total peripheral lymphocyte count, and total cholesterol concentration. Serum ALB concentration is affected not only by nutritional status but also by changes in body fluid volume, such as dehydration, fluid retention, and chronic disease‐induced inflammatory responses.^32^, ^33^ Therefore, the CONUT score is more easily influenced by outside interference. In contrast, SMI is a highly objective measurement based on the use of CT scans to measure body composition, with a reported measurement error of approximately 1.4%.^26^ Moreover, SMI markers are relatively stable, and rapid fluctuations in skeletal muscle mass are unlikely to occur over a short period of time. This objectivity and stability are conducive to correctly predicting patient prognosis. In contrast to Yoshida et al's study,^18^ there were no statistically significant differences in 5‐year OS and RFS between patients with light malnutrition and those with moderate or severe malnutrition. This outcome suggests that the ability of the CONUT score to determine the long‐term survival of patients with light and moderate or severe malnutrition remains unproven. This finding might also be associated with the small number of patients with severe malnutrition (n = 58, 10.9%) in our study. These possible explanations require further research to confirm. Nevertheless, SMI is currently a better predictor than the CONUT score of long‐term survival after radical surgery for patients with GC.

We acknowledge several potential limitations of the present study. First, 235 patients were excluded from the study because they had no available abdominal CT data, which might have resulted in selection bias. Second, the design was retrospective, and the cases were obtained from a single center; therefore, the findings must be confirmed in prospective studies. Nevertheless, for the first time, this study compared the prognostic value of preoperative SMI and the CONUT score to predict long‐term outcomes in GC, revealing that SMI was a more stable and objective predictor than the CONUT score.

## 5 CONCLUSION

Skeletal muscle index based on preoperative CT images is superior to the CONUT score in terms of prognostic value in GC after RG. Therefore, preoperative SMI should be included in preoperative risk assessment, although this conclusion must be confirmed by a large‐scale, prospective validation study.

## CONFLICT OF INTEREST

There are no conflict of interests or financial ties to disclose from any authors.
