# Supplementary figures and images for: Preoperative skeletal muscle index vs the controlling nutritional status score: Which is a better objective predictor of long‐term survival for gastric cancer patients after radical gastrectomy?
Source: Cancer Med. 2018 Jun 28;7(8):3537–47. doi: 10.1002/cam4.1548 (PMC6089186; doi:10.1002/cam4.1548)

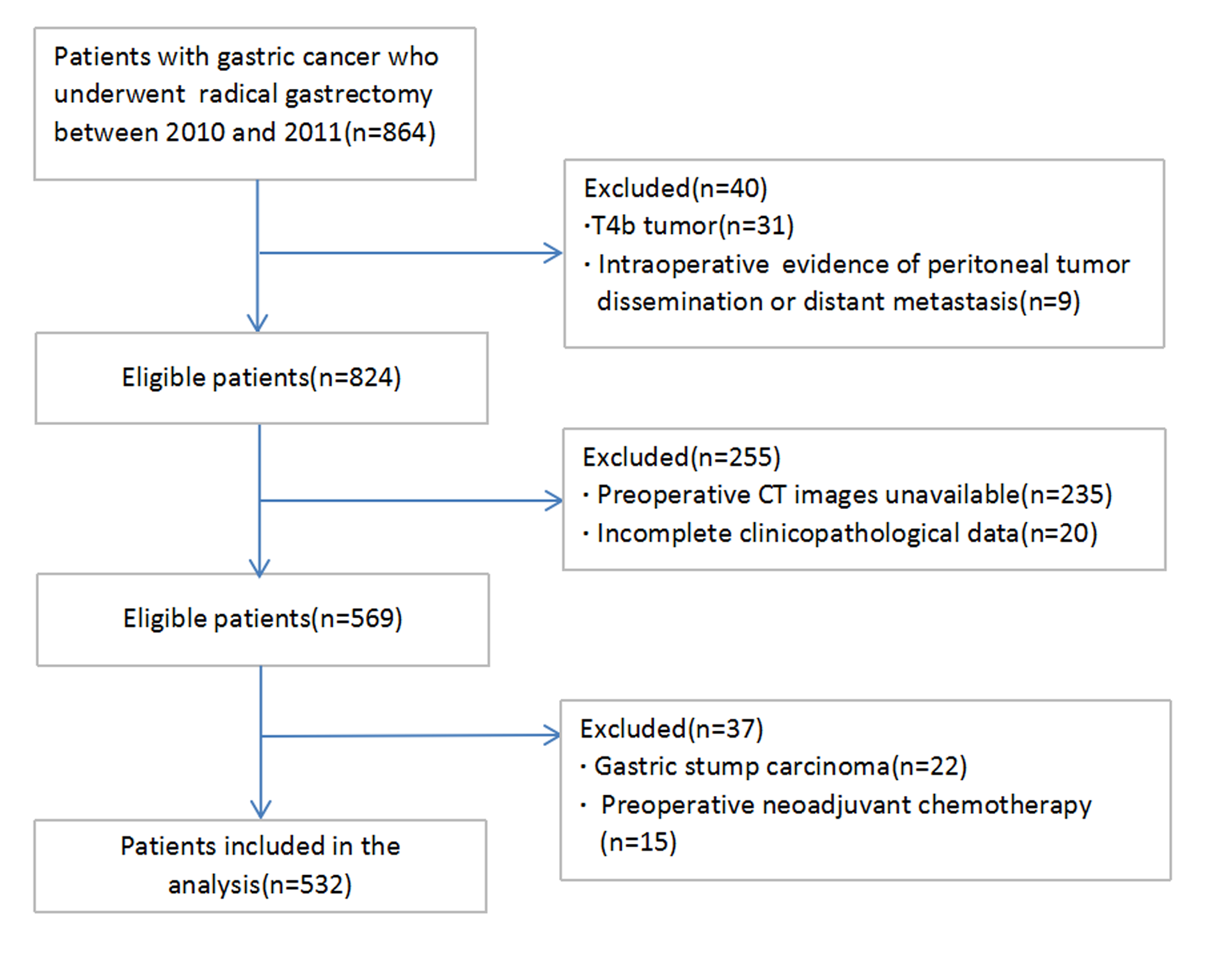

Supplement: Supplementary file 1 [file CAM4-7-3537-s001.tif]
