# Supplementary material for: Preoperative skeletal muscle index vs the controlling nutritional status score: Which is a better objective predictor of long‐term survival for gastric cancer patients after radical gastrectomy?
Source: Cancer Med. 2018 Jun 28;7(8):3537–47. doi: 10.1002/cam4.1548 (PMC6089186; doi:10.1002/cam4.1548)
Supplement: Supplementary file 2 [file CAM4-7-3537-s002.doc]

**Supplemental Table 1**  Assessment of undernutrition degree based on the CONUT.

| **Parameters** | **COUNT** | | | |
| --- | --- | --- | --- | --- |
|  | **Normal** | **Light** | **Moderate** | **Severe** |
| **Serum albumin (g/dL）** | 3.5-4.5 | 3.0-3.49 | 2.5-2.9 | ＜2.5 |
| **Alb score** | 1 | 2 | 4 | 6 |
| **Total lymphocyte (count/mm^3^)** | ≥1600 | 1200-1599 | 800-1199 | ＜800 |
| **TLC score** | 0 | 1 | 2 | 3 |
| **Total cholesterol(mg/dl)** | ＞180 | 140-180 | 100-139 | ＜100 |
| **T-cho score** | 0 | 1 | 2 | 3 |
| **COUNT score (total)** | 0-1 | 2-4 | 5-8 | 9-12 |
| **Assessment** | Normal | Light | Moderate | Severe |

The CONUT score is calculated as the sum of the Alb score, TLC score, and T-cho score.

Alb: albumin; TLC: total lymphocyte; T-cho: total cholesterol
